# Anti-dengue Vaccines: From Development to Clinical Trials

**DOI:** 10.3389/fimmu.2020.01252

**Published:** 2020-06-18

**Authors:** Josilene Ramos Pinheiro-Michelsen, Rayane da Silva Oliveira Souza, Itana Vivian Rocha Santana, Patrícia de Souza da Silva, Erick Carvalho Mendez, Wilson Barros Luiz, Jaime Henrique Amorim

**Affiliations:** ^1^Laboratório de Agentes Infecciosos e Vetores, Centro das Ciências Biológicas e da Saúde, Universidade Federal do Oeste da Bahia, Barreiras, Brazil; ^2^Programa de Pós-graduação em Biologia e Biotecnologia de Microrganismos, Universidade Estadual de Santa Cruz, Barreiras, Brazil

**Keywords:** dengue, vaccine development, pre-clinical tests, clinical trials, countries

## Abstract

*Dengue Virus* (DENV) is an arbovirus (arthropod-borne virus). Four serotypes of DENV are responsible for the infectious disease called dengue that annually affects nearly 400 million people worldwide. Although there is only one vaccine formulation licensed for use in humans, there are other vaccine formulations under development that apply different strategies. In this review, we present information about anti-dengue vaccine formulations regarding development, pre-clinical tests, and clinical trials. The improvement in vaccine development against dengue is much needed, but it should be considered that the correlate of protection is still uncertain. Neutralizing antibodies have been proposed as a correlate of protection, but this ignores the key role of T-cell mediated immunity in controlling DENV infection. It is important to confirm the accurate correlate of protection against DENV infection, and also to have other anti-dengue vaccine formulations licensed for use.

## Introduction

*Dengue Virus* (DENV) is an arbovirus (arthropod-borne virus) transmitted to humans by mosquitoes of the *Aedes* genus (1). There are four serotypes of DENV (DENV1–4) that belong to *Flavivirus* genus of the Flaviviridae family. These are enveloped viruses with an icosahedral capsid and a genome composed of single stranded RNA of positive polarity, which encodes a single polyprotein that gives rise to three structural proteins (C, capsid; prM, membrane; E, envelope) and seven non-structural proteins: NS1, NS2A, NS2B, NS3, NS4A, NS4B, and NS5 (2). The four serotypes of DENV cause a disease called dengue (3) that annually affects nearly 400 million people worldwide (4). Dengue is a fast growing public health problem caused by many factors such as increased urbanization, population growth, increasing migration, and international travel, as well as the difficulties of effective vector control. Together, these factors contribute to the spread of the disease (5).

The World Health Organization (WHO) has highlighted the development of a safe and effective vaccine against the four serotypes of DENV as a priority. However, the vaccine development is challenging because of a limited understanding of the viral pathogenesis. A pathological phenomenon known as antibody-dependent enhancement (ADE) is well-reported in literature. Antibodies generated in response to a first infection by a specific serotype are able to recognize another serotype at a second infection. However, they are not specific and, therefore, neutralization of viral particles is not effective. The antigen-antibody complex is recognized by Fc-γ receptors-bearing phagocytic cells, which facilitates viral entry and provides an enhanced replicative capacity for the virus (2).

There are currently six vaccine formulations at different stages of development with only one licensed for use. Most of these vaccines are primarily based on the envelope proteins prM and E, which are believed to induce protective immune responses in humans (6–9). However, it is very important to consider that the human immune response to DENV is dominated by highly cross-reactive antibodies endowed with neutralizing and enhancing activity (8, 10). This leads us to question the importance of epitopes contained in envelope proteins with regard to the generation of a protective immune response (11). Another very important point to note in the development of anti-dengue vaccines, especially attenuated tetravalent live vaccines, is that the replication of all four DENV serotypes must be balanced, as dominant epitopes may interfere with replication of non-dominant serotypes. This phenomenon may result in preferential antibody response to dominant strains, which could lead to a severe disease on dengue challenge (12).

**Figure 1:**
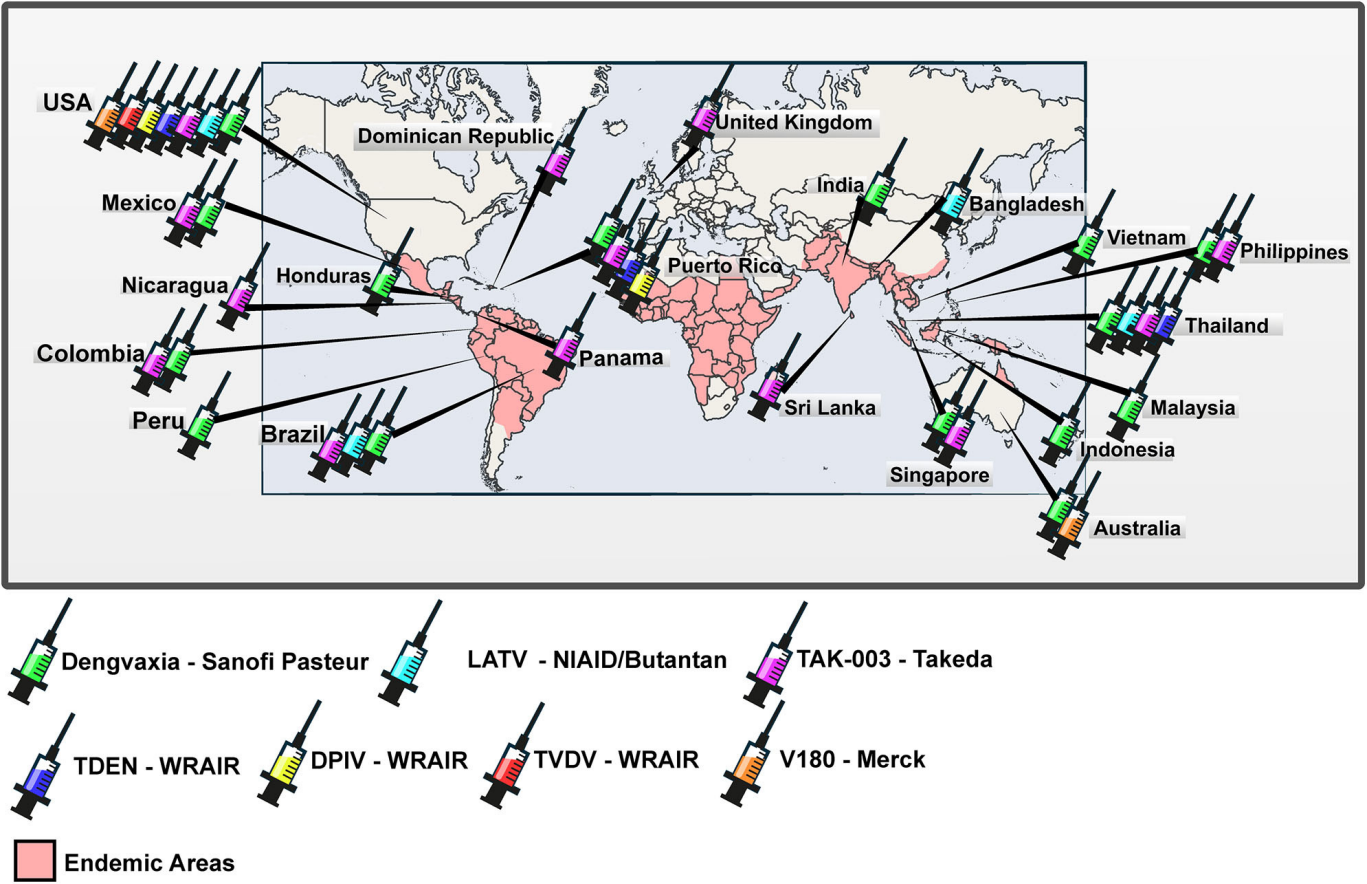
Worldwide distribution of clinical trials of anti-dengue vaccines. World map with the representation of areas in which dengue is endemic and countries in which clinical trials registered at ClinicalTrials.gov were carried out or are being carried out.

In this review, we present information regarding the development, preclinical and clinical trials of anti-dengue vaccine formulations. We included only vaccine formulations with at least one published result of clinical trial. Positive and negative points of vaccine formulations discussed in the text are presented in Table 1. In addition, phase I, II, and III clinical trials that have been carried out in several endemic and non-endemic countries worldwide are indicated in the map (Figure 1).

**Table 1:** Pros and cons of the seven anti-dengue vaccines registered at ClinicalTrials.gov (accessed until March 31, 2020).

**Vaccine candidate and manufacturer**	**Phase^a^**	**Countries^b^**	**Age range**	***n*^c^**	**Main results of clinical trials**	**References**
**Dengvaxia**^®^ Sanofi Pasteur	4	20	From 9 months to 60 years	48,387	POSITIVE POINTS: Tested under independent phase III clinical trials. Licensed for use in humans in 20 countries and available in 10 countries across Latin America and Asia. NEGATIVE POINTS: Does not contain the non-structural proteins of DENV. Presents low protective efficacy, especially for children and increases their risk of hospitalization. Immunization schedule composed of three doses schedule. Cannot be administered in *Flavivírus-naïve people*. Suitable for ages ranging from 9 to 45 years.	(13–48)
**LATV** NIAID/Butantan/Merck	3	4	From 12 to 70 years	18,300	POSITIVE POINTS: Shown to be safe. Contains all structural and non-structural proteins of DENV. Immunization schedule composed of a single dose. Protected all human volunteers in challenge trial. Shown to be safe and immunogenic in children and adults, regardless contact with flaviviruses. NEGATIVE POINTS: Requires an increased amount of DENV2 for a balanced seroconversion. Results of protective efficacy not yet released.	(49–59)
**TAK-003** Takeda	3	15	From 2 to 60 years	27,500	POSITIVE POINTS: Shown to induce seroconversion with neutralizing antibodies to all four DENV serotypes. Shown to induce cellular immune response. Shown to be safe and immunogenic for children and adults, regardless contact with flaviviruses. Primary efficacy data showing vaccine efficacy of 80.2%. NEGATIVE POINTS: Induces low titles of antibodies to DENV3. Immunization schedule composed of two doses.	(60–64)
**TDEN** U.S. Army Medical Research and Materiel Command	2	3	From 12 months to 45 years	907	POSITIVE POINTS: Acceptable reactogenicity and safety profile in children and adults. Induced low viremia. Induced seroconversion to the four DENV serotypes after animmunization schedule composed of two doses. Most of the generalsymptoms reported were classified asmild or moderate and transient. NEGATIVE POINTS: Viremia associated with arthralgia, headache, fatigue, muscle aches, and pain behind the eyes on day 8 post dose, and detected in four vaccinees of F17/Pre.	(65–68)
**DPIV** U.S. Army Medical Research and Materiel Command	1	2	From 18 to 39 years	200	POSITIVE POINTS: Acceptable safety profile. Induced high and balanced neutralizing antibody responses to all four DENV serotypes in *Flavivírus*-naive healthy adult subjects. Induced a tetravalent immune response when co-administered with a live attenuated vaccine. NEGATIVE POINTS: Few clinical studies using small samples of volunteers. Neutralizing antibody titres decrease over time. Although most adverse events were of mild intensity, there were some of moderate intensity.	(69, 70)
**TVDV** U.S. Army Medical Research and Materiel Command	1	1	From 18 to 50 years	40	POSITIVE POINTS: Shown to be safe and well-tolerated. Elicited predominantly anti-DENV T-cell IFN-γ responses. NEGATIVE POINTS: Presents only E and PrM proteins. High amounts of vaccine antigen needed. Adjuvant needed. Three-dose immunization regimen. Differences in seroconversion rates among DENV.	(71)
**V180** Merck	1	2	From 18 to 49 years	98	POSITIVE POINTS: Shown to be safe. Immunogenic with low amounts of vaccine antigens. NEGATIVE POINTS: Depends on adjuvants to be immunogenic. Three-dose immunization regimen.	(72, 73)

a *Phase of clinical trial*.

b *Countries where clinical trials have been performed or are being performed*.

c *Approximate number of individuals already enrolled in clinical trials*.

## Main Body

### Dengvaxia^®^

**Figure 2:**
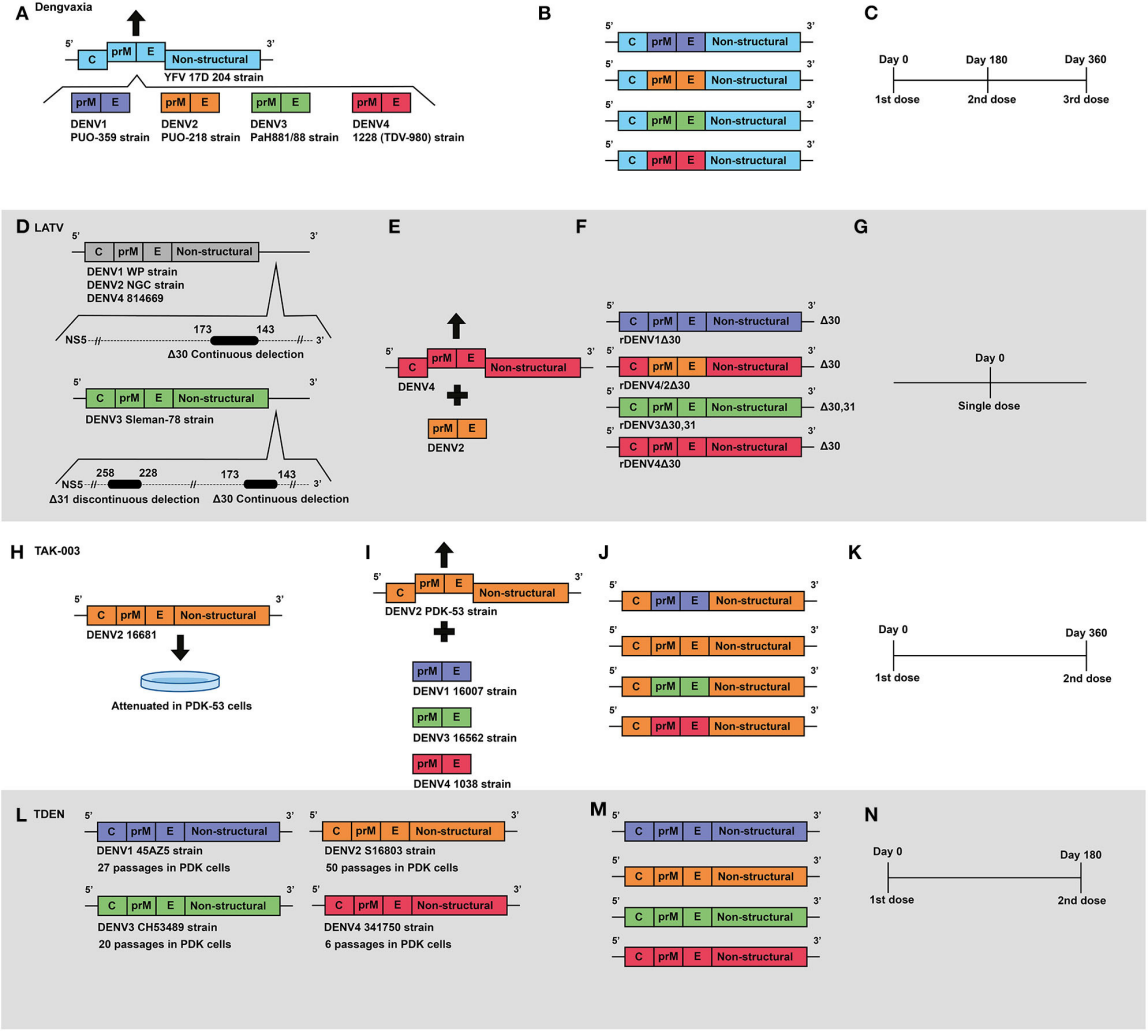
Development of live attenuated vaccines by Sanofi, NIAID/Butantan, Takeda and U.S. Army Medical Research and Materiel Command. **(A)** Development of the Sanofi vaccine. The YFV 17D vaccine virus was used as a backbone for the construction of chimeric viruses, replacing its envelope protein-encoding genes with those from wild-type DENV serotypes: DENV1 (PUO-359) DENV2 (PUO-218) DENV3 (PaH881/88) DENV4 (1228). **(B)** Representation of genetic construction of the four DENV vaccine viruses, which compose the current Dengvaxia^®^ tetravalent vaccine. **(C)** Current Sanofi vaccine immunization regimen, based on a three-dose schedule. **(D)** Development of NIAID/Butantan vaccine. Live attenuated vaccine viruses were generated by the introduction of continuous 30 nucleotide deletions in the 3′UTR) in DENV1 (WP), DENV2 (NGC), and DENV4 (814669). For the DENV3 Slemen-78 strain, in addition to the original 30 nucleotide deletion, an additional 31 nucleotide discontinuous deletion was carried out at the 3′UTR. **(E)** The DENV-2 component of NIAID/Butantan vaccine was generated by chimerization. prM and E genes from DENV2 (NGC) strain were introduced in replacement of those ofrDEN4Δ30. **(F)** Representation of the four DENV1–4 vaccine constructs, which compose the current NIAID/Butantan tetravalent vaccine formulations TV003 and TV005. **(G)** Current vaccination regimen of NIAID/Butantan vaccine. **(H)** Development of Takeda's live attenuated recombinant vaccine, in which the strain 16681 DENV2 was attenuated by 53 serial passages in PDK cells. **(I)** Takeda DENV1, DENV3, and DENV4 vaccine antigens were developed by recombining the DENV1 (16007), DENV3 (16562), and DENV4 (1036) strains with the DENV2 PDK53 vaccine virus. **(J)** Representation of genetic composition of vaccine viruses contained in Takeda's vaccine. **(K)** Current Takeda's vaccine immunization regimen. **(L)** Development of TDEN vaccine formulations, in which strains 45AZ5 (DENV1), S16802 (DENV2), CH53489 (DENV3), and 341750 (DENV4) were attenuated with serial passages in PDK cells. **(M)** Representation of genetic composition of vaccine viruses contained in the current TDEN vaccine formulation. **(N)** TDEN F17 and F19 vaccine formulations were administered in two doses 180 days apart in clinical trials.

The anti-dengue vaccine of Sanofi Pasteur is a live, attenuated, and tetravalent recombinant vaccine called ChimeriVax™, Dengvaxia^®^ or CYD-TDV (Chimeric yellow fever-dengue—tetravalent dengue vaccine)—here, vaccine's names will be used according to the history of vaccine development, as shown in Table 2. It was initially developed by the National Institutes of Health (NIH) and the University of St. Louis (74, 75). The vaccine formulation consists of chimeric viruses constructed by using infectious clone technology. Genomic sequences encoding the pre-membrane (prM) protein and the envelope glycoprotein (E) of the 17D strain of *Yellow fever virus* (YFV) were replaced by those of each of the four serotypes of DENV (76) (Figures 2A,B). The 17D strain of YFV has been used for more than 6 decades as a vaccine. It was selected as a backbone for chimerization because of its safety, rapid onset and duration of immunity (77). The DENV strains used in the construction of chimeras were: DENV1 PUO359 strain, isolated in Thailand in 1980, and named ChimeriVax-D1; DENV2 PUO-218 strain, isolated from a child during the 1980 epidemic in Bangkok, and named ChimeriVax-D2; DENV3 strain PaH881/88, isolated in Thailand in 1988, and named ChimeriVax-D3; and DENV4 1228 strain, isolated in 1978 in Indonesia, and named ChimeriVax-D4 (77–79).

**Table 2:** Timeline of vaccine development.

**Current name^a^**	**Year^b^**	**Name^c^**	**Valence**	**Vaccine formulation**	**Developer/manufacturer**	**Evaluation^d^**
Dengvaxia^®^	1991	Type 1-2 and 4 Chimeric Virus	Monovalent	Chimeric viruses YH/DEN1-2 and 4	NIAID^e^	*In vitro*
	2000	ChimeriVax-D2	Monovalent	Chimeric virus YF/DEN2	St. Louis University^f^	*In vivo* (animal)
	2001	ChimeriVax-™	Mono/tetravalent	Chimeric viruses YF/DEN1-4	Acambis, Inc.	*In vivo* (animal)
	2006	ChimeriVax™-DEN2	Monovalent	Chimeric virus YF/DEN2	Sanofi Pasteur/Acambis, Inc.	*In vivo* (phase I trial)
	2010	TDV	Tetravalent	Chimeric viruses YF/DEN1-4	Sanofi Pasteur	*In vivo* (phase I trial)
	2011	CYD-TDV	Tetravalent	Chimeric viruses YF/DEN1-4	Sanofi Pasteur	*In vivo* (phase II–III trials)
	2015	Dengvaxia^®^	Tetravalent	Chimeric viruses YF/DEN1-4	Sanofi Pasteur	Licensed
LATV	1996	rDEN1Δ30 rDENV2/4Δ30 rDENV3Δ30/31-7164 rDENV4Δ30	Monovalent	Genetically attenuated virus (deletion) Chimeric virus DENV2/rDEN2/4Δ30 Genetically attenuated virus (deletion) Genetically attenuated virus (deletion)	NIAID	*In vitro* and *in vivo* (Animal and phase I trial)
	2003	LATV Formulations: (TV001, TV002, TV003, TV004)	Tetravalent	Three genetically attenuated viruses and one chimeric virus	NIAID and Butantan^g^	*In vivo* (phase I–III)
TAK-003	1987	DENV2 PDK-53	Monovalent	Virus attenuated with passages in PDK cells	University Mahidol	*In vitro* and *in vivo* (Animal and phase I trial)
	2003	DENV2 PDK-53/1 DENV2 PDK-53 DENV2/3 DENV2/4	Monovalent	Chimeric viruses DENV2 PDK-53/DENV1,3, or 4	University of Texas and Inviragen, Inc.	*In vitro* and *in vivo* (Animal)
	2011	DENVax1-4	Monovalent	Chimeric viruses DENV2 PDK-53/DENV1,3, or 4	Inviragen, Inc.	*In vivo* (animal)
	2015	TDV	Tetravalent	Chimeric viruses DENV2 PDK-53/DENV1,3, or 4	Takeda	*In vivo* (phase I-III trials)
	2019	TAK-003	Tetravalent	Chimeric viruses DENV2 PDK-53/DENV1,3, or 4	Takeda	*In vivo* (phase III trial)
TDEN	2003	DENV (serotype 1,2,3, and 4)	Mono/Tetravalent	Viruses attenuated with passages in PDK cells	WRAIR^h^	*In vitro* and *in vivo* (animal)
	2006	TDEN (Formulations: F17 and F19)	Tetravalent	Virus attenuated with passages in PDK cells	WRAIR and GlaxoSmithKline	*In vivo* (phase I-II trials)
DPIV	1995	PIV	Monovalent	Purified-inactivated virus (DENV2), aluminum hydroxide as an adjuvant	WRAIR	*In vivo* (animal)
	2010	TPIV	Tetravalent	Purified-inactivated viruses (DENV1–4), aluminum hydroxide AS01, AS03, or AS04 as adjuvants	NMRC^i^, WRAIR	*In vivo* (animal)
	2015	TDENVPIV	Tetravalent	Purified-inactivated viruses (DENV1–4), aluminum hydroxide AS01, AS03, or AS04 as adjuvants	WRAIR and GlaxoSmithKline	*In vivo* (animal)
	2017	DPIV	Tetravalent	Purified-inactivated viruses (DENV1–4), aluminum hydroxide AS01, AS03, or AS04 as adjuvants	WRAIR, GlaxoSmithKline and Fiocruz^j^	*In vivo* (phase I trial)
TVDV	1997	DEN-2	Monovalent	DNA vaccine based on pRM and 92% of E protein of DENV2	NMRC and Vical Inc.	*In vivo* (animal)
	2000	DIME100	Monovalent	DNA vaccine based on prM and 100% of protein E of DENV1	WRAIR	*In vivo* (animal)
	2003	1040D2ME-LAMP	Monovalent	Chimeric DNA vaccine based on prM and E proteins of DENV2 and the mouse lysosome-associated membrane protein (LAMP).	NMRC	*In vivo* (animal)
	2006	DEN-3	Monovalent	Nucleic acid vaccine DEN3: prM and protein E complete	NMRC	*In vivo* (animal)
	2012	TVDV	Tetravalent	DNA vaccine based on prM and E protein coding sequences cloned in the VR1012 plasmid co-administered with VAXFECTIN^®^ as an adjuvant.	U.S. AMRDC^k^, WRAIR, NMRC and Vical Inc.	*In vivo* (animal and phase I trial)
V180	2010	DEN80E	Mono/Tetravalent	Recombinant proteins based on prM and 80% of the E protein of DENV1–4 combined with different adjuvants.	Hawaii Biotech, Inc., WRAIR	*In vivo* (animal)
	2018	V180	Tetravalent	Recombinant proteins based on prM and 80% of the E protein of DENV1–4 combined with different adjuvants.	Merck & Co., Inc.	*In vivo* (phase I trial)

a *Current name of the vaccine formulation*;

b *Year in which the name was used for the first time*;

c *Name of vaccine formulation considering the year of development step*;

d *Vaccine formulations were evaluated in vitro and in vivo. In vivo assays involve pre-clinical tests in animals models and/or phase I, II and III clinical trials*;

e *National Institute of Allergy and Infectious Diseases, National Institutes of Health*;

f *Department of Molecular Microbiology and Immunology, St. Louis University Medical School, St. Louis*;

g *Butantan Institute, São Paulo, Brazil*;

h *Walter Reed Army Institute of Research*;

i *Naval Medical Research Center*;

j *Oswaldo Cruz Foundation*;

k *U.S. Army Medical Research and Development Command*.

Reports of development of monovalent vaccine formulations were published in 2000 (77) for DENV2 and in 2001 for DENV1, DENV3, and DENV4 (78). In preclinical studies, ChimeriVax™ viruses were shown to have similar growth with regard to wild-type DENV, as evaluated in human myeloid dendritic cells (DCs) and in three hepatic cell lines (HepG2, Huh7, and THLE-3). In contrast with YFV 17D strain, chimeric viruses were shown to be non-hepatotropic, as shown by their lack of growth in human liver cells (80). In addition, ChimeriVax viruses were shown to be highly attenuated for *Aedes albopictus* and *Aedes aegypti* mosquitoes in terms of infection and dissemination (81). Moreover, non-human primates (NHP) were shown to develop protective humoral immune response after a single dose immunization regimen capable of controlling levels of viremia (76). Importantly, chimeric viruses were shown to be non-neurovirulent in mice and lead to viremia at similar levels of the YFV 17D vaccine strain in NHP (78).

Clinical trials with the vaccine were carried out in North and South America, Asia, and Oceania (see Figure 1), in order to cover populations with different flavivirus infections and vaccination histories (13). Trials of phase I, II, and III involved more than 40,000 volunteers from 16 countries (see Table 1) and evaluated safety, immunogenicity, and protective efficacy. ChimeriVax™-DEN2 presented a safety level similar to that of YF-VAX^®^. In addition, previous immunity to YFV did not interfere with ChimeriVax™-DEN2 immunization and a long-lasting immunity with high serum levels of cross-neutralizing antibodies was observed to all four DENV serotypes after immunization (82). In Mexico, the tetravalent vaccine was subjected to a phase I clinical trial based on an immunization regimen of three doses given in children and adults. The vaccine formulation, named at that time as TDV (tetravalent dengue vaccine), was shown to be safe and capable of eliciting a neutralizing antibody response against the four serotypes of DENV (83). In another phase I trial with flavivirus-naive adults, seroconversion to DENV1 in a two-dose regimen was lower (92%) compared to a regimen based on three doses, which reached 100% seroconversion to all serotypes of DENV (84). In Philippines, immunogenicity was compared in immunization regimens of two or three doses, and 85% of the volunteers seroconverted to all DENV serotypes, regardless of the immunization regimen (85). The safety and immunogenicity profiles of TDV continued to be evaluated in phase II trials.

The vaccination induced a neutralizing antibody response against all DENV serotypes and was well-tolerated in children and adolescents in phase II trials carried out in Brazil (14) and in Colombia, Honduras, Mexico, and Puerto Rico (15). Relevantly, it was shown that prior exposure to YFV or monovalent dengue vaccines does not produce adverse effects or any other safety issues. Conversely, such prior exposure contributes to an increased immunogenicity of the vaccine formulation (16). Moreover, a three-dose regimen had a good safety profile in 2–11 years old Peruvian children with a history of YFV vaccination and elicited robust antibody responses that were balanced against the four DENV serotypes (17). Other phase II clinical trials supported the evaluation of the vaccine formulation (named as CYD-TDV at that time) under phase IIb as well as phase III trials (18–20). Further phase II studies registered at ClinicalTrials.gov have been developed to assess the safety and immunogenicity of the vaccine.

As promising results were achieved in phase I and phase II clinical trials, Sanofi Pasteur carried out studies aiming to evaluate protective efficacy based on an immunization regimen composed of three doses of CYD-TDV with intervals of 6 months. In a phase IIb trial carried out with Thai children from 4 to 11 years of age, protective efficacy was shown to be 30.2% **(**Confidence Interval—CI 95%: 13.4–56.6). Moreover, protective efficacy against DENV2 was shown to be 9.2% (CI 95%: 75–51.3) (21). However, in phase III clinical trials carried out in children from Latin America and Asia, CYD-TDV protective efficacy was shown to be of 60.8% (CI 95%: 52.0–68.0) and of 56.5% (CI 95%: 43.8–66.4), respectively (22, 31). In such trials, protective efficacy against DENV2 was increased by 35% (CI 95%: 9.2–61.0). Long-term evaluations have shown that CYD's efficiency profile does not apply for all age groups.

In a study involving more than 35,000 children aging from 2 to 16 years old, the combined efficacy rate for symptomatic dengue during the first 25 months after immunization regimen was 60.3% (CI 95%: 55.7–64.5) for all participants, 65.6% (CI 95%: 60.7–69.9) for persons aged 9 years or older and 44.6% (CI 95%: 31.6–55.0) for children under 9 years of age. In addition to the limited protective efficacy, vaccinated patients younger than 9 years old were at a higher risk of hospitalization due to dengue infection. Therefore, vaccination was restricted to those over 9 years of age (23). Importantly, studies have shown that the quality of the neutralizing antibodies induced by CYD-TDV varies depending on the serotype of DENV and the previous immunological status. Individuals without pre-existing DENV-specific immunity developed neutralizing antibodies to all 4 serotypes, and DENV4 was primarily neutralized by type-specific antibodies, whereas DENV1, DENV2, and DENV3 were primarily neutralized by cross-reactive antibodies (47). However, pre-existing immunity to DENV ensured the development of higher levels of neutralizing antibodies. Recent vaccine trials have demonstrated efficacy against virologically confirmed symptomatic dengue, with higher estimates of efficacy for DENV3 and DENV4, and moderate estimates of efficacy for DENV1 and DENV2 (24). In addition, it was found that hospitalization rates due to severe dengue were significantly higher in dengue naive children who received Dengvaxia (26). Thus, the administration of this vaccine was restricted to seropositive people (26, 86).

Several studies were initiated aiming to evaluate the efficacy of CYD-TDV when co-administered with other vaccine antigens and adjuvants. Co-administration of YF-VAX^®^ vaccine with CYD-TDV had no relevant impact on the immunogenicity or safety profile of the YF-VAX^®^ (25). Other trials registered at ClinicalTrials.gov are currently underway, in order to assess the immunogenicity and safety of a CYD-TDV concomitantly administered with Adacel^®^ (diphtheria, tetanus, and acellular pertussis adult vaccine—also called Tdap) in healthy subjects aged from 9 to 60 years in Philippines (NCT02992418), Gardasil in healthy subjects aged from 9 to 13 years in Malaysia (NCT02993757) and Cervarix^®^ in healthy female subjects aged from 9 to 14 years in Mexico (NCT02979535).

Regarding the pattern of immune response elicited by CYD-TDV, neutralizing antibodies were shown to be important. However, in a study carried out with flavivirus-*naive* and flavivirus-immune healthy volunteers immunized with CYD-TDV, there was not triggering of detectable changes in serum pro-inflammatory cytokines. These responses were dominated by DENV4 in *naive* individuals, and were broadened when a booster dose was carried out 4 months after the first dose. A broader response was detected after primary tetravalent immunization in volunteers with pre-existing immunity to DENV1 or DENV2, which was elicited by a prior monovalent live-attenuated DENV immunization. In all three trials presented in the study, the profiles of cellular immune responses elicited were similar, whatever the volunteer's immune status, i.e., an absence of Th2 response, and an IFN-gamma/TNF-alpha ratio dominated by IFN-gamma, for both CD4 and CD8 T cell responses. In addition, an absence of cross-reactivity between YFV or DENV NS3-specific CD8 T cell responses was shown, which allowed the identification of 3 new CD8 epitopes in the YFV NS3 antigen (87).

In view of the promising results obtained in the various clinical studies with CYD-TDV, Mexican authorities granted marketing authorization for Dengvaxia^®^ on December 9, 2015 (Dengvaxia^®^ is the current commercial name of CYD-TDV). Thus, this is the first anti-dengue vaccine to be licensed in the world. Philippines, Brazil, El Salvador, Costa Rica, Paraguay, Guatemala, Peru, Indonesia, Thailand, and Singapore also granted regulatory approval for Dengvaxia^®^ (88). Recently, on December 19, 2018, the European Commission also granted marketing authorization for Dengvaxia^®^, and although it does not contemplate the whole world, it remains the only anti-dengue licensed vaccine so far. The indication of vaccination is restricted to individuals living in endemic regions and aged between 9 to 45 years, in a three-dose vaccination regimen (Figure 2C), with the exception of Paraguay, which extended the upper age limit to 60 years (88).

### LATV

The Laboratory of Infectious Diseases (LID) of the National Institute of Allergy and Infectious Diseases (NIAID) developed a live attenuated tetravalent vaccine for dengue (LATV) (89)—here, vaccine's names will be used according to the history of vaccine development, as shown in Table 2. Using recombinant DNA technology, LID originally developed DENV4 attenuated virus by removing nucleotides from position 172–143 of the 3′ untranslated region (3′UTR) of the strain 814669 Dominica 1981 (89) (Figure 2D). The rDEN4Δ30 mutant exhibited reduced infectivity and replicated less efficiently in LLC-MK2 cells when compared to the wild type virus (89). It also exhibited reduced growth in C6/36 cells, induced antibody response equivalent to a wild-type DENV1 (89), and was shown to have restricted ability to infect and disseminate in the midgut of *Aedes aegypti* mosquitoes (90). In clinical studies rDEN4Δ30 was shown to be a safe and immunogenic vaccine virus (91, 92).

The deletion of 30 nucleotides was also carried out in a homologous region of DENV1, strain Western Pacific (WP), originating the rDEN1Δ30 (93). Such virus was shown to be attenuated in rhesus monkeys at levels similar to rDEN4Δ30 vaccine virus (93). All monkeys inoculated with rDEN1Δ30 were completely protected against a challenge carried out with a wild-type DENV1. In addition, attenuation was also shown in HuH-7-SCID mice (93). The replication in *Toxorhynchites splendense* mosquitoes was shown to be restricted (93). And in the clinical trials it was shown to be safe and immunogenic (94, 95).

For the development of the DENV2 vaccine virus, two methods of virus attenuation (nucleotide deletion added to chimerization) were used (96, 97). Chimerization was carried out by using the rDEN4Δ30 infectious clone as a backbone. Two chimeric viruses were generated by replacing the region encoding the structural proteins in the rDEN4Δ30 backbone with the homologous part from DENV2 strain NGC: (i) rDEN2/4Δ30 (ME) (Figures 2D,E), with replacement of membrane (M) and envelope (E) glycoproteins and (ii) rDEN2/4Δ30 (CME), with replacement of capsid (C) protein, in addition to M and E. (97). The two chimeras were shown to be highly attenuated in SCID-HuH-7 mice, mosquitoes, and rhesus monkeys (97). Due to the satisfactory results obtained in the pre-clinical tests (94, 97), the rDEN2/4′ (ME) virus was evaluated in phase I clinical trials, in which it was shown to be safe and immunogenic (49).

Several vaccine candidates were proposed for the DENV3 (98). The first of them was developed by chimerization, with replacement of prM and E proteins coding regions of the rDEN4 and rDEN4Δ30 virus with those of DENV3 (Sleman/78 strain). Two chimeric viruses were generated: rDEN3/4 (ME) and rDEN3/4Δ30 (ME) (98). Effective attenuation was shown for the two chimeric viruses in SCIDHUH-7 mice and rhesus monkeys. Although they were shown to elicit significant titers of neutralizing antibodies, they produced detectable viremia in monkeys, and the infectivity of the viruses in the midgut of *Ae. Aegypti* was similar to the wild type DENV3 (98). The second approach was the deletion of 30 nucleotides in the 3′UTR region of the Sleman/78 strain, originating the mutant rDEN3Δ30 virus (98). All NHP immunized with rDEN3Δ30 were shown to produce neutralizing antibody in titers equivalent to the wild-type DENV3, which guaranteed effective protection after challenge. However, attenuation was not seen in any tested animal model (98). The rDEN3/4Δ30 (ME) chimeric virus was chosen for further evaluation in clinical trials, but seroconversion was observed in only 25% of volunteers (99). In view of the low attenuation of rDEN3Δ30 in SCID-HuH-7 mice and NHP, new strategies were employed (100). Further deletions were carried out (100) in the original mutation 30 (nt173–143) of the 3'UTR region (98), and generated nine mutant viruses that were considered viable (100). The mutant virus rDEN3Δ30/31, which includes the original mutation and an additional non-continuous deletion of 31 nt (258–228) (Figure 2D), presented complete loss of replication in C6/36 cells, in addition to a robust replication in Vero cells. Moreover, it exhibited reduced replication in *Toxorynchites* mosquitoes and low infection rates in rhesus monkeys, without detectable viremia. The rDEN3Δ30/31 mutant virus was also shown to elicit strong neutralizing antibody response in rhesus monkeys, capable of conferring protection against a challenge with DENV3 (100). Finally, it induced a seroconversion of 90% in volunteers in a clinical trial (50).

A phase I clinical trial evaluated the safety and immunogenicity of monovalent vaccine candidates (rDEN1Δ30, rDEN2/4Δ30, and rDEN3Δ30/31-7164) (101). All three vaccines were well-tolerated by volunteers, with mild and short-term adverse events, with only a short-lived, low-level viremia (<100 PFU/mL of blood). The DEN1Δ30 and DEN3Δ30/31-7164 viruses induced 90% of seroconversion, while the rDENV2/4Δ30 chimeric virus induced an immune response with low levels of specific antibodies (101).

In view of the results obtained in the pre-clinical and clinical trials with the monovalent vaccine formulations of DENV, live attenuated tetravalent vaccine (LATV) formulations were proposed to be evaluated in phase I, II, and III clinical trials in order to determine safety, reactogenicity, infectivity, immunogenicity and protective efficacy profiles. In a phase I clinical trial, four LATV formulations were evaluated: **TV001** (rDEN1Δ30, rDEN2/4Δ30, rDEN3-3′D4Δ30, and rDEN4Δ30) TV002 (rDEN1Δ30, rDEN2/4Δ30, rDEN3-3′D4Δ30 rDEN4Δ30-200,201); TV003 (rDEN1Δ30, rDEN2/4Δ30, rDEN3Δ30/31, and rDEN4Δ30) (see Figure 2F), and TV004 (DEN1Δ30, rDEN2/4Δ30, rDEN3Δ30/31, rDEN4Δ30-200,201) (102). All vaccines were shown to be well-tolerated, and 93% of all local adverse events induced were of mild severity. In addition, eruption occurred in 64.2% of volunteers (102), in a similar way with regard to reactions observed with the monovalent vaccines (101). Low-level viremia occurred frequently, but with less detection for DEN2/4Δ30 and DEN4Δ30-200, 201, and none of the participants developed fever (102). Moreover, antibody titers against each monovalent component were relatively balanced, inducing seroconversion rates of 50 to 100%. rDEN2/4Δ30 induced the lowest rates, ranging from 50 to 65%. The most balanced antibody response was achieved when rDEN3Δ30/31 and rDEN4Δ30 were mixed with rDEN1Δ30 and rDEN2/4Δ30 (TV003), inducing seroconversion in 100% for DENV1 and DENV4, and 50 and 85% for DENV2 and DENV3, respectively (102). In a clinical trial with TV003, 48 healthy adults received two doses of vaccine or placebo given 12 months apart. A single dose of TV003 elicited sterilizing immunity to all 4 serotypes for at least 1 year in 80% of vaccines. Vaccine viremia was not detected in any vaccine following the second dose (51).

Phase I/II clinical trials were performed with naive flavivirus adults to demonstrate the safety and immunogenicity of two vaccine formulations (52). The TV003 vaccine formulation was previously shown to elicit better seroconversion rates (90%) (102), and the TV005 vaccine formulation. The TV005 is similar to TV003, but with an increased amount of the DENV2 vaccine component from 10^3^ to 10^4^ PFU (52). In a single dose regimen, both, TV003 and TV005 induced seroconversion to all DENV serotypes. However, the elicitation of tetravalent immune response was increased from 74% with TV003 to 90% with TV005 (52). In addition, the specific immune response to DENV2 also increased from 76% with TV003 to 97% with TV005 administration. Relevantly, both, the first dose and the second doses (given 6 months after) were well-tolerated. Importantly, a significant increase in antibody titers was not observed after the booster at 6 months (52).

Although less effective in inducing seroconversion to DENV2 if compared to TV005, the protective efficacy of TV003 was assessed in a human challenge experiments with rDEN2Δ30 (DENV2) in DENV*-naive* individuals (53). Initially, rDEN2Δ30 was developed from strain Tonga/74 of DENV2 as a vaccine candidate, with a deletion of 30 nucleotides in the 3'UTR region. However, it was shown to be infectious in *Toxorynchites* mosquitoes, and only slightly attenuated in rhesus monkeys (103). Moreover, it was shown to be infective and to induce signs of dengue in healthy volunteers (54). Thus, it was abandoned as a vaccine antigen, but was proposed to be used in challenge experiments (53). In this phase I clinical trial, all 24 volunteers immunized with TV003 seroconverted to DENV2, DENV3, and DENV4, while 91.7% seroconverted to DENV1. And relevantly, all volunteers were protected after a challenge with rDEN2Δ30 (103).

As evidenced, there have been numerous obstacles to the development of a safe and effective vaccine against dengue, and currently two single dose vaccines (see Figure 2G) are being evaluated in Phase I, II, and III clinical trials. Four phase I clinical trials involving TV003 and TV005 vaccine formulations are currently registered in ClinicalTrials.gov. In human challenge experiments, both TV003 (NCT03416036) and TV005 (NCT02317900 and NCT02873260, respectively) have been evaluated against DENV2 and DENV3. TV005 is also being evaluated regarding its safety and immunogenicity in flavivirus-*naive* adults (NCT02879266). Moreover, two evaluations of phase II trials (Table 1), in important dengue endemic countries, are ongoing in order to assess safety and immunogenicity of TV003 and TV005. They are tested in adults, adolescents and children in Bangladesh (TV005 NCT02678455) and Thailand (TV003 NCT02332733). In addition, Butantan Institute (Brazil) is carrying out phase II and phase III clinical studies in Brazil with the TV003 vaccine formulation, in a partnership with NIAID. Trials are registered at ClinicalTrials.gov with codes NCT01696422 and NCT02406729. Results regarding protective efficacy are being awaited.

### TAK-003

Takeda Pharmaceutical Company Limited is leading the development of a live attenuated tetravalent vaccine named as Tak-003, that is based on an attenuated virus and chimeric viruses constructed using recombinant DNA technology (104)—here, vaccine's names will be used according to the history of vaccine development, as shown in Table 2. The initial studies with the vaccine were developed in the 1980s, at the Mahidol University, in Thailand. With the aim of attenuating the viral strains DENV1 16007, DENV2 16681, and DENV4 1036, passages in primary dog kidney cells (PDK) were carried out. And the DENV3 viral strain 16562 was attenuated with passages in green monkey kidney (GMK) and fetal rhesus lung (FRhL2) cells (55, 105–109). The vaccine candidates presented no evidence of neurovirulence in mice and low viremia in monkeys. However, when tested in humans the vaccine viruses caused systemic reactions consistent with a dengue-like syndrome and the study was stopped early to avoid further risks (110). Nevertheless, the vaccine candidate DENV2 (16681) PDK 53 was shown to be promising when tested as a monovalent formulation (106, 111), and was used as a backbone in the constructs of the chimeric vaccine viruses of DENV1, DENV3, and DENV4 (112, 113) (Figures 2H,I).

The first chimera to be developed was based on the recombination of genes coding for non-structural proteins of the DENV2 PDK-53 virus with those coding for envelope proteins (prM and E) of the DENV1 16007 virus (112). The resulting DENV2/DENV1 vaccine virus preserved DENV2 PDK-53 attenuation markers, such as temperature sensitivity in LLC-MK2 cells, low replication in C6/36 cells and attenuation in mice. It was also shown to elicit higher titers of neutralizing antibodies against the DENV1, when compared to the DENV1 PDK-13 vaccine virus. In continuity, the constructs of vaccine candidates for DENV1, DENV3, and DENV4 were evaluated with different recombinations between wild type viruses and PDK-attenuated vaccine viruses (113). Nine chimeric viruses containing DENV1 (16007), DENV3 (16562), or DENV4 (1036) wild-type pre-membrane (prM) and envelope (E) genes within the DENV2 (16681) and the two genetic variants (PDK53-E and PDK53-V) of the DENV2 PDK-53 vaccine virus were generated. As a result, the DENV2 PDK-53 vaccine virus was shown to be an interesting vector for the development of live, attenuated flavivirus vaccines. In addition, the DENV2 PDK-53 was shown to replicate uniformly even when recombined and has the potential to induce a balanced immunity against all four serotypes of DENV (113). Thus, the University of Texas and Inviragen carried out studies to assess the safety and genetic stability of DENV2-PDK-53 vaccine virus and chimeric vaccine viruses DENV2/1, DENV2/3, and DENV2/4. It was shown that the vaccine virus maintained the previously defined safety characteristics, including the three main genetic attenuation *loci*, temperature sensitivity in mammalian cells, very low infection and dissemination in *Aedes aegypti* and reduction of neurovirulence in mice (114). Therefore, the DENV2-PDK-53 was successfully used to produce the candidate tetravalent vaccine, which is currently being tested in clinical trials in humans (60–62, 115).

The DENVax vaccine, which contains the vaccine components DENV2-PDK-53 (DENVax2), DENV2/1 (DENVax1), DENV2/3 (DENVax3), and DENV2/4 (DENVax4) (Figure 2J), was subjected to pre-clinical tests carried out with mice and NHP in order to evaluate its safety, immunogenicity and protective capacity (116, 117). In mice, the monovalent formulations (DENVax1, DENVax2, DENVax3, and DENVax4) were shown to be safe and to elicit robust neutralizing antibody responses with protective capacity (116). In Cynomolgus monkeys, the vaccine was well-tolerated, with low levels of viremia, and induced neutralizing antibodies against the four DENV serotypes. All the immunized animals were protected from challenges carried out with DENV3 and DENV4, and a lower dose of the DENVax formulation partially protected the animals from challenges with DENV1 or DENV2 (117).

Takeda conducted a clinical trial in Colombia to evaluate the safety and immunogenicity of the tetravalent vaccine (DENVax) in flavivirus-naïve young and healthy adults. In addition, both formulations (low and high dose) were well-tolerated and induced neutralizing antibody responses to all four serotypes. However, antibody titers were lower for serotypes 3 and 4 (60). The vaccine, now renamed as TDV, was tested in flavivirus-naïve young and healthy adults with versions of the vaccine formulation differing in the amount of the DENV4 antigen. All formulations were well-tolerated and there were no reports of serious adverse effects (AE). The seroconversion rates were 84–100% for DENV1, 96–100% for DENV2, 83–100% for DENV3 and 33–77% for DENV4. In addition, more than 80% of participants in each group seroconverted to at least three serotypes of DENV (104). In Phase I and II clinical trials, TDV was well-tolerated in children and adults aged from 1.5 to 45 years, regardless of previous exposure to DENV. Moreover, seroconversion with production of neutralizing antibodies for all four DENV serotypes, as well as reactive T-cell mediated responses -which are required for a broad protection against dengue- were detected (115).

Phase II clinical trials were organized in dengue endemic countries (Dominican Republic, Panama and the Philippines) in order to determine the safety and immunogenicity of TDV. Healthy participants aged from 2 to 17 years, received one or two doses of the vaccine (Figure 2K), with intervals of 3 months (61, 62). The reactogenicity profiles were acceptable, with neutralizing antibodies being elicited against all DENV serotypes, regardless of previous exposure to DENV. In addition, the trials demonstrated that a second dose of TDV induces increased immunogenicity against DENV3 and DENV4 in flavivirus-naïve persons, suggesting that a two-dose regimen of TDV induces a more robust humoral immune response. Recently, Takeda presented primary efficacy data from part 1 of an ongoing phase 3 randomized trial of a tetravalent dengue vaccine candidate (named as TAK-003) in regions of Asia and Latin America in which the disease is endemic. The immunization regimen was composed of two doses of TAK-003 in children and adolescents 4–16 years of age (see Figure 2K). As a result, they showed an overall vaccine efficacy in the safety population of 80.9% (118). In addition, a 95.4% efficacy against dengue leading to hospitalization was reported (118).

### TDEN F17/F19

The Walter Reed Army Institute of Research (WRAIR) developed a live-attenuated, tetravalent dengue (TDEN) vaccine in collaboration with GlaxoSmithKline (GSK)—here, vaccine's names will be used according to the history of vaccine development, as shown in Table 2. The vaccine formulation consists of viruses obtained from natural infections that were isolated in C6/36 cells and then attenuated with serial passages in PDK cells. The 341750 strain of DENV4 was the first TDEN vaccine virus to be attenuated with 20 passages, in the early beginning of the 1990's. Thereafter, the following viral strains were also attenuated in PDK cells: 45AZ5 strain of DENV1, with 20 passages; S16803 strain of DENV2, with 50 passages; and CH53489 strain of DENV3, with 20 passages (see Figures 2L,M). Vaccine bulks were prepared with propagation of viruses in FrhL cells. All steps of development and production of vaccine formulation were evaluated regarding presence of contaminating agents. Pre-clinical studies carried out in Rhesus monkeys with both, monovalent and tetravalent versions of TDEN, previously named as DENV vaccine, revealed they are safe (119).

Aiming to identify the best dosage of vaccine components, 16 tetravalent formulations differing in their vaccine virus content were subjected to a phase I clinical trial with flavivirus-naïve adults. As a result, the formulations 13 and 14 were selected for further evaluations (120). A new formulation (F17pre) was developed aiming to optimize the neutralizing antibody response. It was prepared with 27 and six passages of DENV1 and DENV4 in PDK cells, respectively. F17pre, F13 and F14 were then subjected to a phase II clinical trial with two doses given with a 6-month interval to flavivirus-naïve adults. Few grade 3 AE were reported. In addition, the seroconversions to DENV serotypes elicited after a second dose were less variable in F17pre group: 69, 100, 81, and 94% to 1, 2, 3, and 4 serotypes, respectively (121). The F17pre vaccine was selected to be the precursor of two formulations: F17 and F19, both re-derived by propagation in FrhL cells. In a phase II clinical trial, F17 and F19 formulations elicited 37.9 and 40% of tetravalent seroconversion, respectively (65). Moreover, F19 was shown to be the safest formulation: 15.4% of the subjects reported injection site pain, whereas the rate for F17 was near 35%, and 100% of flavivirus-primed subjects seroconverted to all four DENV antibodies when immunized with F19, against 97.1% for F17 (122).

In a randomized phase II clinical trial conducted in Puerto Rico, the seroconversion rates for all DENV serotypes revealed a similar immunogenic profile for both formulations. The F17 elicited higher titers of neutralizing antibodies after a second dose. Moreover, F17 elicited 100% seroconversion to all serotypes in primed subjects, while F19 induced 98.4% of seroconversion for DENV2, DENV3, and DENV4. Among the non-primed volunteers, the values were variable for both formulations. The lowest rate of seroconversion after immunization with F17 was observed for DENV3: 92.7%. However, F19 was shown to induce seroconversion in 78% of non-primed volunteers (66). Despite the better immunogenic profile of F17 with regard to F19, both vaccines were shown to be safe and immunogenic in primed and non-primed volunteers. In order to assess the safety and immunogenicity profiles of the TDEN F17 vaccine, a pilot study was conducted in 6–7 years old Thai children who were shown to be naïve for DENV1–4 and *Japanese encephalitis virus* (JEV) (67). Results of the study showed that the vaccine is safe and immunogenic and contributed to the advance to a phase I/II trial in infants aged 12–15 months. Volunteers tolerated the vaccine well without any AE, and after the second dose, 85.7% of them developed an immune response to at least three DENV serotypes. In addition, 53.6% of immunized volunteers seroconverted to the four serotypes of DENV (68). To date, the vaccine has been shown to be safe and immunogenic in volunteers ranging from 12 months to 50 years of age, when administered in a two-dose regimen (Figure 2N). However, a study enrolled in the ClinicalTrials.gov (NCT01843621) aims to understand more about TDEN F17, through a 5-year follow-up of the volunteers who received the two doses of the vaccine in the previously cited trial (67). It will also assess the safety and immunogenicity of a third dose administered 1 year after the second dose, and its results may contribute to elucidate the schedule of administration for this candidate vaccine.

### DPIV

The tetravalent dengue purified inactivated vaccine (DPIV) was developed by the Walter Reed Army Institute of Research (WRAIR) and is manufactured by the WRAIR Pilot Bioproduction Facility and adjuvanted by GlaxoSmithKline (GSK) adjuvant systems—here, vaccine's names will be used according to the history of vaccine development, as shown in Table 2. The vaccine development started in 1995, when a DENV2 (S16803 strain) was isolated from a patient in C6/36 cells and then propagated in Vero cells by three passages. A master seed was prepared at passage two and a production seed at passage three. Difficulties in virus propagation, as well as the apparent antigenic instability after formalin inactivation, led the inactivated vaccines to have their viability questioned. Nevertheless, the purified inactivated virus (PIV) was shown to be safe when administered to mice, which developed significant neutralizing antibodies titers and 100% of seroconversion after the second dose (123). Further, the PIV was shown to be safe and immunogenic when administered to Rhesus monkeys. In addition, an immunization regimen based on two doses administered 3 months apart was shown to be safe, without exhibition of redness or swelling at the vaccination sites. Moreover, neutralizing antibody titers were reported in 37 of 39 vaccinated animals (124).

**Figure 3:**
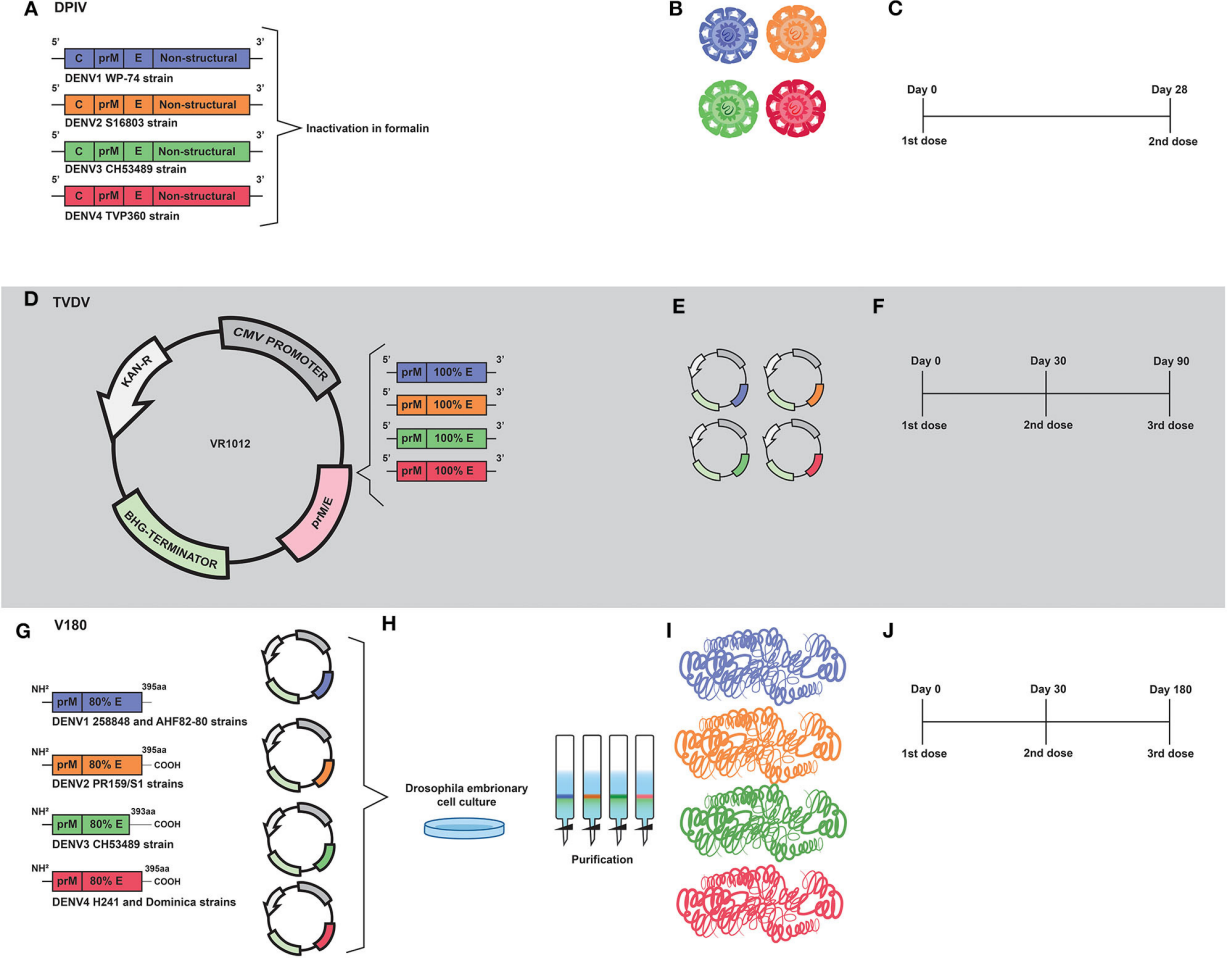
Development of inactivated (DPIV) and DNA (TVDV) vaccines by the U.S. Army Medical Research and Materiel Command; and the development of the V180 subunit vaccine by Merck. **(A)** DPIV is composed of formalin-inactivated viral particles of DENV1 WP-74, DENV2 S16803, DENV3 CH53489, and DENV4 TVP360 strains. **(B)** Representation of the four vaccine constructs which compose the current DPIV vaccine formulation. **(C)** Representation of DPIV immunization regimen, which consists of two doses 28 days apart. **(D)** Development of the tetravalent DNA vaccine (TVDV). Genetic constructs encoding prM and E proteins of DENV1 (west Pac74), DENV2 (New Guine C strain), DENV3 (Asian strain) and DENV4 were cloned into Plasmid VR1012. **(E)** Representation of the genetic constructs which are the vaccine antigens of the current TVDV vaccine. **(F)** The TVDV immunization regimen consists of three doses administered on days 0, 30, and 60. **(G)** Development of a tetravalent vaccine based on prM and 80% (ectodomain) of the E protein (V180). Coding regions of prM and E protein ectodomain of DENV1 (258848 and AHF82-80 strains), DENV2 (PR159/51), DENV33 (CHS3489), and DENV4 (H242 Dominica) were cloned into expression plasmids. **(H)** Drosophila Schneider-2 (S2) cells were used for expression of prM/E. Recombinant vaccine antigens were purified by immunoaffinity chromatography. **(I)** Representation of proteins that compose the current V180 vaccine formulation. **(J)** The immunization regimen used in the V180 clinical trial consisted of three doses administered on days 0, 30, and 180.

Years later, a tetravalent formulation based on inactivated viruses named as TPIV (tetravalent purified-inactivated virus), was developed and subjected to immunization assays in Rhesus monkeys using three other virus strains derived from human isolates: West pac 74 for DENV1, CH53489 for DENV-3, and TVP360 for DENV-4; in addition to the previously mentioned strain of DENV2. Viruses propagated in Vero cells were subjected to sucrose gradient ultracentrifugation purification approach, followed by formalin inactivation and addition of 0.01% (v/v) alum (aluminum hydroxide) (125) as an adjuvant (Figures 3A,B). The immunization regimen was composed of a first dose of TPIV followed by a booster dose of a tetravalent live attenuated vaccine (TLAV). Such a vaccine regimen elicited virus neutralizing antibodies. The TPIV/TLAV combination afforded complete protection against DENV 3 challenge at month 8. In a second experiment, priming with TPIV elicited neutralizing antibodies against all four serotypes of DENV. After challenge with each one of the four DENV serotypes, vaccinated animals exhibited no viremia but showed anamnestic antibody responses to the challenge viruses.

Renamed as TDEN PIV, the vaccine formulation based on inactivated viruses was subjected to an evaluation regarding adjuvants options in Rhesus monkeys. As a result, most of adjuvants tested helped to prevent viremia after challenge, with the DENV1 and DENV2 administered at 40 and 32 weeks post-dose 2, respectively (126). Afterwards, a monovalent vaccine formulation based on the DENV1 West pac 74 strain was prepared by nine passages in Rhesus monkey lung (FrhL) diploid cell cultures followed by three passages in Vero cells. Such a vaccine was subjected to a phase I clinical trial in a non-endemic area of the United States, and was shown to be safe and moderately immunogenic. Volunteers were immunized in a two-dose regimen; 28 days apart. A low number of subjects reported mild pain or tenderness after the first injection. Seroconversion reached 100% in volunteers 2 weeks after the second dose (127). Such results guided vaccine developers to replace alum with AS01_E_ (3-O-desacylcinomonophosphoryl lipid A) and AS03_B_ (Oil-in-water emulsion) adjuvant systems, aiming to overcome problems with immunogenicity caused by inactivation. Their combination has been demonstrated to induce early mobilization of neutrophils and monocytes (128, 129).

More recently, the tetravalent vaccine formulation, named as DPIV and adjuvanted by AS01E/AS03B, was evaluated in a phase I clinical trial with 100 healthy adults, also in the United States. Volunteers were immunized in a regimen of two doses, with an interval of 28 days (Figure 3C). In agreement with the previous trial, pain, redness and swelling were frequently reported. Neutralizing antibody responses against all four DENV serotypes were balanced in the dengue-naive adults, but decreased over time (69). Moreover, DPIV was shown to induce high titers of neutralizing antibodies in a phase I clinical trial carried out in Puerto Rico. The best results were achieved when AS03_B_ was used as an adjuvant (70).

The humoral responses observed with administration of vaccine formulations based on inactivated viruses were robust, though it is still unknown whether DPIV is able to provide long lasting immunity. Despite presenting a balanced antibody responses against the four serotypes, the inactivated nature of vaccine antigens may still generate obstacles regarding immune response to non-structural proteins. As described above, only envelope and capsid proteins were targeted by the immune system after immunization regimens with DPIV. The vaccine was unable to control DENV infection in challenge assays carried out with immunized rhesus macaques. Importantly, increased levels of viremia, aspartate transaminase, IL-10, IL-18 and IFN-γ, and reduced levels of IL-12 were detected in immunized NHP, indicating that vaccination may have triggered antibody-dependent enhancement of DENV infection (130).

### TVDV

A tetravalent DNA vaccine against dengue (TVDV) was developed by the U.S. Army Medical Research and Materiel Command—here, vaccine's names will be used according to the history of vaccine development, as shown in Table 2. It is based on the genes that code for the entire pre-membrane (prM) and envelope (E) proteins and is currently being tested in a Phase I clinical trial (71, 131, 132).

The monovalent version of the DNA vaccine against DENV1, initially named as DIME100, is derived from West Pacific strain 74 consisting of prM and E genes cloned into the plasmid VR1012 (133) (Figures 3D,E). Such vaccine formulation was shown to be immunogenic and to protect Rhesus monkeys under challenge assays. It was also shown to be immunogenic in phase I clinical trials (131).

It was first shown to be immunogenic and to protect mice under challenge assays with DENV2 when co-administered with CpG motifs (134, 135) (Figures 3D,E). Then, the original construct was modified by replacing the DENV2 transmembrane and cytoplasmic sequences with those of the mouse lysosome-associated membrane protein. At that time, the vaccine was renamed as 1040D2ME-LAMP (136). Such modification strategy targets MHC class II compartment and elicits long-lasting neutralizing antibodies.

The DENV3 antigen was derived from an Asian viral strain and is composed of prM and E, which were cloned in VR1012 plasmid (Figures 3D,E). Preclinical tests carried out with *Aotusnancymae* showed that the DENV3 monoclonal vaccine elicits neutralizing antibody and moderate levels of specific IgG. In addition, it was also shown to confer partial protection in challenge assays (137).

The genetic construction of DENV4 is similar to that of DENV3 (Figures 3D,E). However, there is a lack of reports showing results of preclinical tests in its monovalent form. Nevertheless, a tetravalent vaccine (TVDV) formulation initially based on shuffled constructs was developed. Three formulations were initially proposed: sA and sC (based on prM and E) and sB (encoding envelope protein ectodomain only). Such constructs were administered to Rhesus monkey and only those immunized with sA and sC produced antibodies to the four serotypes (138).

In a subsequent study, Vaxfectin^®^ was used as an adjuvant aiming to improve immunogenicity of a tetravalent vaccine formulation based on non-chimeric genetic constructs. These constructs were prepared by combining equal amounts of monovalent plasmid DNA vaccines that encode the pre-membrane (prM) and envelope (E) genes of DENV1–4 cloned into the VR1012 plasmid. In an immunization regimen composed by three doses (Figure 3F), the use of Vaxfectin^®^ resulted in a significant increase in anti-DENV neutralizing antibody responses against DENV1, DENV2, and DENV3. In addition, co-administration of TVDV adjuvanted with Vaxfectin^®^ diminished the time of viremia in challenged animals (131). The tetravalent formulation containing Vaxfectin^®^ was also tested in New Zealand white rabbits for evaluation of safety and immunogenicity and animals receiving two doses of the vaccine formulation were shown to seroconvert to the four serotypes of DENV (132). Finally, in a phase I clinical trial (NCT01502358) carried out with 40 flavivirus-naïve volunteers, TVDV was shown to be safe and well-tolerated and elicited dose-dependent anti-DENV T-cell IFN-γ responses (71).

### V180

A vaccine formulation based on recombinant forms of the DENV envelope glycoprotein was initially developed by Hawaii biotech and named as DEN-80 due to its composition, based on the ectodomains of the E protein of each DENV serotype, which correspond to 80% of the whole protein- here, vaccine's names will be used according to the history of vaccine development, as shown in Table 2. Currently, the product is being developed by Merck and the vaccine formulation is adjuvanted by ISCOMATRIX™ (139).

Genetic constructs of the tetravalent vaccine formulation V180 were prepared by RT-PCR- based amplification of viral sequences coding for prM and truncated E proteins, with subsequent digestion and cloning into pMttΔXho vector (139) (derived from pMttPA and pMttbns plasmids). The following viral sources were used for RT-PCR: DENV1 strain 258848 and DENV1 Thailand AHF82-80 (GenBank accession number D00502); DENV2 strain PR159/S1 (140, 141); DENV3 strains CH53489 and D3H87 (142) and DENV4 strains H241 and Dominica (143) (Figure 3G). Drosophila Schneider-2 (S2) cells were used for the expression of the prM/E sequences with secretion of the vaccine antigen for each serotype. Finally, the recombinant vaccine antigens were purified by immunoaffinity chromatography (139) (Figures 3H,I).

In preclinical tests carried out with mice and non-human primates (NHP), the monovalent vaccine formulation composed by ISCOMATRIX adjuvant and DEN2-80E was shown to elicit neutralizing antibodies and to confer protective immunity under lethal challenge assays using the virulent DENV2 strains New Guinea C and S16803. The vaccine formulation was shown to elicit a Th1 profile of cellular immune response with detection of memory cells 6 months after the last immunization (139).

Such positive results regarding immunogenicity were attributed to the high quality of the recombinant proteins, in addition to the use of ISCOMATRIX™ as an adjuvant, which contributed to the achievement of protective immunity with low amounts of vaccine antigen per dose (139, 144). In addition, the tetravalent version of the vaccine formulation, named as V180, was shown to induce higher levels of neutralizing antibodies with an immunization regimen based on doses given at days 0, 30, and 180 (72) (see Figure 3J). Moreover, clinical trials NCT00936429, NCT01477580, showed that V180 induced moderate levels of neutralizing antibodies in human volunteers, with 85.7% seroconversion. It is important to stress that immunogenicity of the vaccine formulation depends on the adjuvant. Conclusion of clinical validation of the vaccine formulation is required in order to define if it is safe and effective.

## Discussion

The advance in vaccine development against dengue is welcome. There is to date one vaccine formulation licensed for use in countries in which the disease is endemic and two other vaccine formulations being tested at phase III clinical trials. However, neutralizing antibodies have been assumed as the main correlate of protection. This is questionable and ignores the key role of T-cell mediated immunity in controlling DENV infection. Dengvaxia^®^ is the most tested anti-dengue vaccine, as can be seen in Figure 1. However, as can be seen in Table 1, it is clear that the lack of DENV non-structural proteins led to a low protective efficacy, especially for children. In addition, children had their risk of hospitalization increased by the use of the vaccine. Moreover, the immunization schedule is composed of three doses and the vaccine cannot be administered to *Flaviv*í*rus-naïve* persons. It seems to be a good model to understand that envelope proteins alone are not able to induce protective immunity. This statement is reinforced by the low or lack of protective capacity of vaccine formulations which induce solely humoral immune response against structural proteins. T cell-based immunity is essential in controlling DENV infection and most of the key targets are located at non-structural proteins (145, 146). Fortunately, vaccine formulations which contain both, structural and non-structural proteins are under clinical trials and one of them (TAK-003) showed a relevant protective efficacy of 80.9% (118). Hopefully, other anti-dengue vaccine formulations will show equivalent or higher protective capacity and will be licensed for use in the near future.

## Author Contributions

JP-M carried out bibliographic review, participated in the preparation of figures and tables, and wrote the manuscript. RS carried out the bibliographic review, participated in preparation of figures, and wrote the paper. IS carried out bibliographic review, participated in preparation of figures, and wrote the paper. PS carried out bibliographic review and participated in preparation of figures. EM carried out bibliographic review and wrote the paper. WL carried out bibliographic review and wrote the paper. JA carried out bibliographic review and wrote the paper. All authors contributed to the article and approved the submitted version.

## Conflict of Interest

The authors declare that the research was conducted in the absence of any commercial or financial relationships that could be construed as a potential conflict of interest.
